# Alteration of extracellular matrix proteins in atrophic periodontal ligament of hypofunctional rat molars

**DOI:** 10.1038/s41405-023-00155-7

**Published:** 2023-07-18

**Authors:** Daneeya Na Nan, Vincent Everts, Joao N. Ferreira, Vorapat Trachoo, Thanaphum Osathanon, Nuttha Klincumhom, Prasit Pavasant

**Affiliations:** 1 Center of Excellence in Regenerative Dentistry, Faculty of Dentistry, Chulalongkorn University, Bangkok, Thailand; 2 Department of Anatomy, Faculty of Dentistry, Chulalongkorn University, Bangkok, Thailand; 3 Department of Oral Cell Biology, Faculty of Dentistry, University of Amsterdam and Vrije Universiteit, Amsterdam, The Netherlands; 4 Avatar Biotechnologies for Oral Health and Healthy Longevity Research Unit, Faculty of Dentistry, Chulalongkorn University, Bangkok, Thailand; 5 Department of Oral and Maxillofacial Surgery, Faculty of Dentistry, Chulalongkorn University, Bangkok, Thailand; 6 Dental Stem Cell Biology Research Unit, Faculty of Dentistry, Chulalongkorn University, Bangkok, Thailand

**Keywords:** Periodontitis, Malocclusion

## Abstract

**Objectives:**

The aim of this study was to investigate the effect of mechanical force on possible dynamic changes of the matrix proteins deposition in the PDL upon in vitro mechanical and in vivo occlusal forces in a rat model with hypofunctional conditions.

**Materials and methods:**

Intermittent compressive force (ICF) and shear force (SF) were applied to human periodontal ligament stem cells (PDLSCs). Protein expression of collagen I and POSTN was analyzed by western blot technique. To establish an in vivo model, rat maxillary molars were extracted to facilitate hypofunction of the periodontal ligament (PDL) tissue of the opposing mandibular molar. The mandibles were collected after 4-, 8-, and 12-weeks post-extraction and used for micro-CT and immunohistochemical analysis.

**Results:**

ICF and SF increased the synthesis of POSTN by human PDLSCs. Histological changes in the hypofunctional teeth revealed a narrowing of the PDL space, along with a decreased amount of collagen I, POSTN, and laminin in perivascular structures compared to the functional contralateral molars.

**Conclusion:**

Our results revealed that loss of occlusal force disrupts deposition of some major matrix proteins in the PDL, underscoring the relevance of mechanical forces in maintaining periodontal tissue homeostasis by modulating ECM composition.

## Introduction

The periodontal ligament (PDL) is a specialized soft tissue of the periodontium that connects the tooth to the surrounding alveolar bone. The main function of PDL is to withstand mechanical forces in the occlusal plane generated from muscular driven movements of mastication, speech, and deglutition. Proper mechanical stimulation is an important factor to maintain the integrity and function of the tooth and periodontium [1]. The lack of contact with an opposing tooth could lead to disuse-type of atrophy in the PDL, eventually resulting in tooth loss [2].

Atrophic changes of the PDL-surrounding hypofunctional teeth include narrowing of the periodontal space, collagen disorganization, and vascular constriction. Finally, it induces dental root and alveolar bone resorption [3, 4]. Despite the extensive literature on hypofunctional teeth and their surrounding periodontal tissues, the effect on the PDL’s extracellular matrix (ECM) components remains elusive.

In vitro mechanical stimulation is known to regulate changes in the ECM protein patterns. For example, static compressive forces upregulate osteopontin, one of the bone’s major non-collagenous proteins that play role in both bone formation and resorption [5]. Intermittent compressive forces (ICF) increase expression of various ECM-associated genes including insulin-like growth factor-1 (*IGF-1*), periostin (*POSTN*), sclerostin (*SOST*), and transforming growth factor-β (*TGF-β*) in PDLSCs [6, 7].

ECM is a diverse and complex structure that aligns PDL cells and provides an environmental niche to maintain their stability and functionality [8]. The ECM can be regarded as a mechanotransduction system being composed of matrix protein components that serve as mechanosensor in response to external forces. The responsible cells then perceive the biophysical signals transmitted via the ECM, which are transduced into intracellular biochemical signals that control many biological and pathological processes such as wound healing, tissue integrity, and ECM remodeling [9].

Collagen type I, a primary structural protein of the ECM, shapes the intrinsic mechanical properties of fibrous connective tissues such as tendons, blood vessels, heart valves, and ligaments [10]. Collagen fibrils are the basic units of force transmission for all connective tissues. Meanwhile, collagenous tissue development and remodeling rely on mechanical force incorporation. Studies revealed a correlation between mechanical stress and the production of extracellular proteases. High magnitude forces applied to human fibroblasts caused at least a 3- to 5-fold increase in expression of matrix metalloproteinase-9 (MMP-9) [11] and a 13-fold increase in MMP-2 [12]. These reports suggested that mechanical forces may modulate collagen turnover by altering collagen degradation. Apart from those external environment influences, internal matrix proteins can be generated intracellularly to govern collagen fibrillogenesis, thereby altering the mechanical characteristics of connective tissues.

Previous studies have shown that periostin (POSTN), a matricellular ECM protein and a member of the fasciclin gene family, also appears to play important roles in the collagen fibrillogenesis process [13]. POSTN is a confined and secreted ECM protein that is abundant in collagen-rich fibrous connective tissues that are frequently exposed to mechanical stresses such as periosteum [14], cardiac valves [15], and the PDL [16]. Numerous findings report the function of POSTN in dental tissues. For instance, expression of POSTN during the interaction between epithelial and mesenchymal tissues during tooth development suggests that POSTN may be crucial for the assembling and positioning of ECM adhesion molecules [17]. The expression of POSTN in tooth and surrounding alveolar bone, according to Suzuki and colleagues [18] indicates that POSTN may be involved in maintaining the integrity of teeth at the interface site of hard-soft tissue. POSTN acts as a point of attachment between cell and matrix, as well as triggers morphogenesis and aids in bearing occlusal biomechanical forces.

Laminin is another ECM protein that is found in the lamina basalis that covers vascular endothelium. Because of this, this protein is frequently subjected to fluid shear stress and serves as a ligand for receptors that are sensitive to mechanical force, such as integrins [19, 20]. In addition, laminin has been demonstrated to facilitate the regulation of shear force (SF) induced β-catenin signaling in colon cancer cells [21]. Hence, it is possible that this protein is involved in responses to mechanical force, particularly induced by SF stimulation. All these findings suggest that ECM remodeling is modulated by specific biomechanical force stimuli in order to maintain connective tissue structural integrity and function.

Thus, we hypothesize that occlusal hypofunction may cause dynamic changes in the periodontal tissue due to a modification of expression and structural reshaping of main ECM components in the PDL. The aim of this study was to investigate dynamic changes of expression of collagen I, POSTN, and laminin in the PDL upon a decrease in biomechanical and occlusal forces driven by hypofunction. The study outcomes will improve our understanding of how biomechanical/occlusal forces can modulate expression and structural integrity of matrix PDL proteins, unveiling the importance of these forces in the maintenance of periodontal homeostasis.

## Material and methods

### Isolation and culture of PDLSCs

The PDL tissue collection and cell isolation technique was approved by the Faculty of Dentistry’s Human Research Ethics Committee at Faculty of Dentistry Chulalongkorn University (HREC-DCU 2022-092). Primary human PDLSCs were isolated from the PDL tissues obtained from three distinct healthy donors. The tissues were collected by gently scraping the periodontal ligament of extracted wisdom teeth following a standard dental treatment plan. The explanted tissue samples were cultured in a high glucose-Dulbecco modified eagle medium (DMEM, Gibco, Waltham, MA) containing 10% fetal bovine serum, 2 mM L-glutamine, penicillin (100 U/ml), streptomycin (100 mg/ml), and amphotericin B (5 mg/ml). They were kept in a humidified environment with 5% CO2 and 37 °C. As PDL cells reached 100% confluence, they were subcultured at a 1:3 ratio. PDLSCs characterization was shown in supplementary materials. Cells from the third through sixth passages and at least three separate donors were utilized in each experiment.

### Mechanical force application

Human PDLSCs were subjected to ICF and SF for 24 h. A compressive force device was used as described previously [22]. Compressive force was created by applying load to the culture media with a moving pestle in a well plate of the same size. A computer-controlled device was utilized to calculate weight and monitor the application of force through a balance positioned beneath the well plate. For intermittent loading, the cycle was adjusted to alternate between a 1-s load and a 2-s release, resulting in about 14 cycles per minute. The strength of the force was fixed at 1.5 g/cm^2^. To create SF in the culture medium a revolving cone-shaped rod encased in a 34 mm-diameter stationary ring was used. Following application, SF was distributed with a magnitude of 5 dyn/cm^2^ at the bottom of the cell culture plate, analyzed by ANSYS FLUENT R16.2 software. The optimal magnitude selection was previously described [23].

### Western blot analysis

Human PDLSCs lysate was extracted using RIPA buffer including a protease inhibitor mixture. Total protein concentration was measured with a BCA protein assay kit (Thermo Fisher Scientific, Waltham, MA). For electrophoresis, each sample containing an identical quantity of protein was placed onto an 8% sodium dodecyl sulfate-polyacrylamide gel. The proteins were put onto a nitrocellulose membrane after electrophoresis. The membrane was incubated with primary antibodies against human POSTN (Abcam, Cambridge, UK) and Type I collagen (Santa Cruz Biotechnology, Dallas, TX) at a working concentration of 100 ng/ml. β-Actin served as an internal control. Following Tris-buffered saline washing, the membrane was incubated with a secondary antibody containing 20 ng/ml HRP (Cell Signaling Technology, Danvers, MA). A substrate for chemiluminescence improved the signals, which were then collected by an image analyzer. The Armersham 680 imaging program was used to measure band density (GE HealthCare, Chicago, IL).

### Animal procedure and induction of occlusal hypofunctional tooth

Nine 8-week-old Wistar male rats were included in the experiment. The animal procedure was approved by the Chulalongkorn University Animal Care and Use Committee (Animal Use Protocol No. 1773019). The sample size for this experiment has been calculated following the formula from [24]. An occlusal hypofunction tooth model was created in mandibular right molars by extracting the first, second, and third right maxillary molars using forceps under intraperitoneal injection of xylazine and ketamine for general anesthesia [25–27]. The rats and all necessary resources including facilities, equipment, and drugs were obtained from the Chulalongkorn University Laboratory Animal Center. Following the surgical procedure, the rats were individually housed and provided with a soft diet. They were granted unrestricted access to drinking water and maintained under controlled conditions of a 12-h light-dark cycle and a constant temperature ranging from 23 to 25 °C. Three rats were euthanized using CO^2^ inhalation for whole mandible extraction at each time point of 4-, 8- and 12-weeks post-extraction of the three maxillary molars, and their mandibles were removed. The time points represented the early, middle, and long term of losing occlusal stimulation. The set of mandibular left molars without opposing tooth extraction (contralateral tooth) was used as the control baseline.

### Micro-computed tomography (micro-CT) and PDL space measurement

The whole maxillary jaw was analyzed using micro-CT imaging. The samples were collected and fixed with 10% (v/v) formaldehyde for 24 h, followed by extensive washing with PBS. All specimens were scanned under 70 kVP, 114 mA, 8 W of X-ray. Total teeth and bone volume was analyzed based on hydroxyapatite (HA) at 1200 mg HA/cc using a micro-CT scanner. The PDL space was measured at 4 positions around the root, starting from the middle left through the apical and ending at the middle right of the root. To measure PDL space, the longest root of the first molar from each section was selected and designated as the center. The measurement was conducted on the center section and on +/−10 sections from the center using micro-CT evaluation program (micro-CT 35 SCANCO MEDICAL, SCANCO Medical AG, Switzerland). At least 3 measurement data from 3 animals were used.

### Histological examination and Immunohistochemistry (IHC)

Following micro-CT analysis, the tooth specimens were decalcified using Surgipath Decalcifier II (Leica Biosystems, Richmond, IL) and processed for paraffin embedding. Sections of 5 μm thickness were cut and stained with Masson’s Trichrome and hematoxylin & eosin (H&E).

For IHC, the sections were deparaffined prior to antigen retrieval using heat-induced epitope retrieval (HIER) technique. The section was immersed in primary antibody targeting to type I collagen, POSTN and laminin (Abcam, Cambridge, UK) followed by AlexaFuor-488 labeled secondary antibody. The antibody was diluted under ratios 1:500, and 1:1000, respectively. DAPI was stained for the nuclei background. Digital and fluorescence images were obtained using an Imager.Z2 Apotome 3 microscope (ZEISS International, Germany). The positively stained cells and blood vessels were quantified by random counting in three different samples obtained from 8-weeks post extraction group (*n* = 3).

### Statistical analysis

Data were plotted using mean and standard deviation. Statistical analysis was conducted using an unpaired Student’s *t*-test and one-way ANOVA followed by Holm-Šídák’s multiple comparisons test. *P* < 0.05 was judged statistically significant. GraphPad Prism9 Software was utilized to conduct the analysis.

## Results

### Mechanical forces stimulate ECM protein production by PDLSCs

**Fig. 1:**
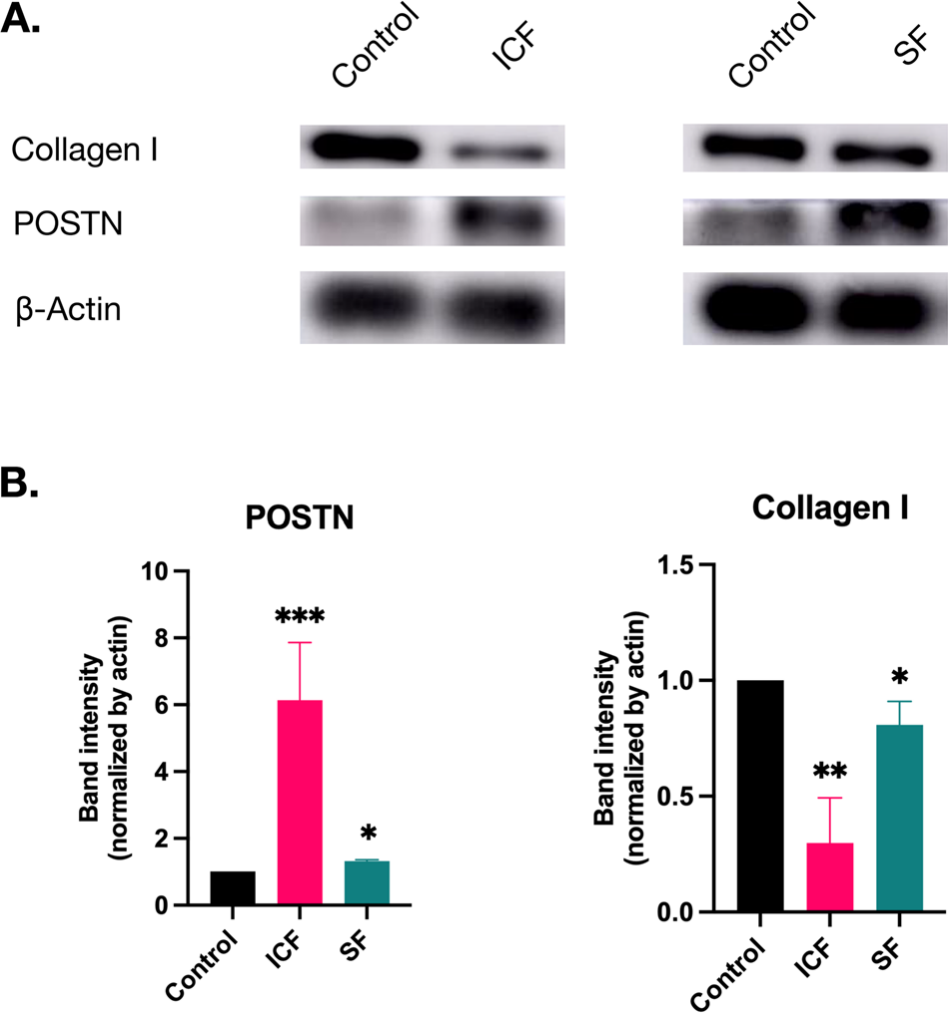
The mechanical forces alter the expression of collagen I and POSTN in human PDLSCs. The PDLSCs were seeded in a 35 cm culture dish and subjected to intermittent compressive force (ICF) and fluid shear force (SF). **A** Western blot analysis of collagen I and POSTN expression. **B** WB band density quantification, β-actin was used as the reference. Data are presented from three biological replicates using PDLSCs obtained from three different donors. *p < 0.05, **p < 0.01, ***p < 0.001 compared to the control.

After 24 h of stimulation, the expression of POSTN protein was significantly increased in both ICF- and SF-induced PDLSCs, whereas the level of collagen I was decreased after both stimulations (Fig. 1A, B). These findings confirm that mechanical forces can alter ECM protein expression in PDLSCs.

### Histological features of PDL in occlusal hypofunction tooth model

**Fig. 2:**
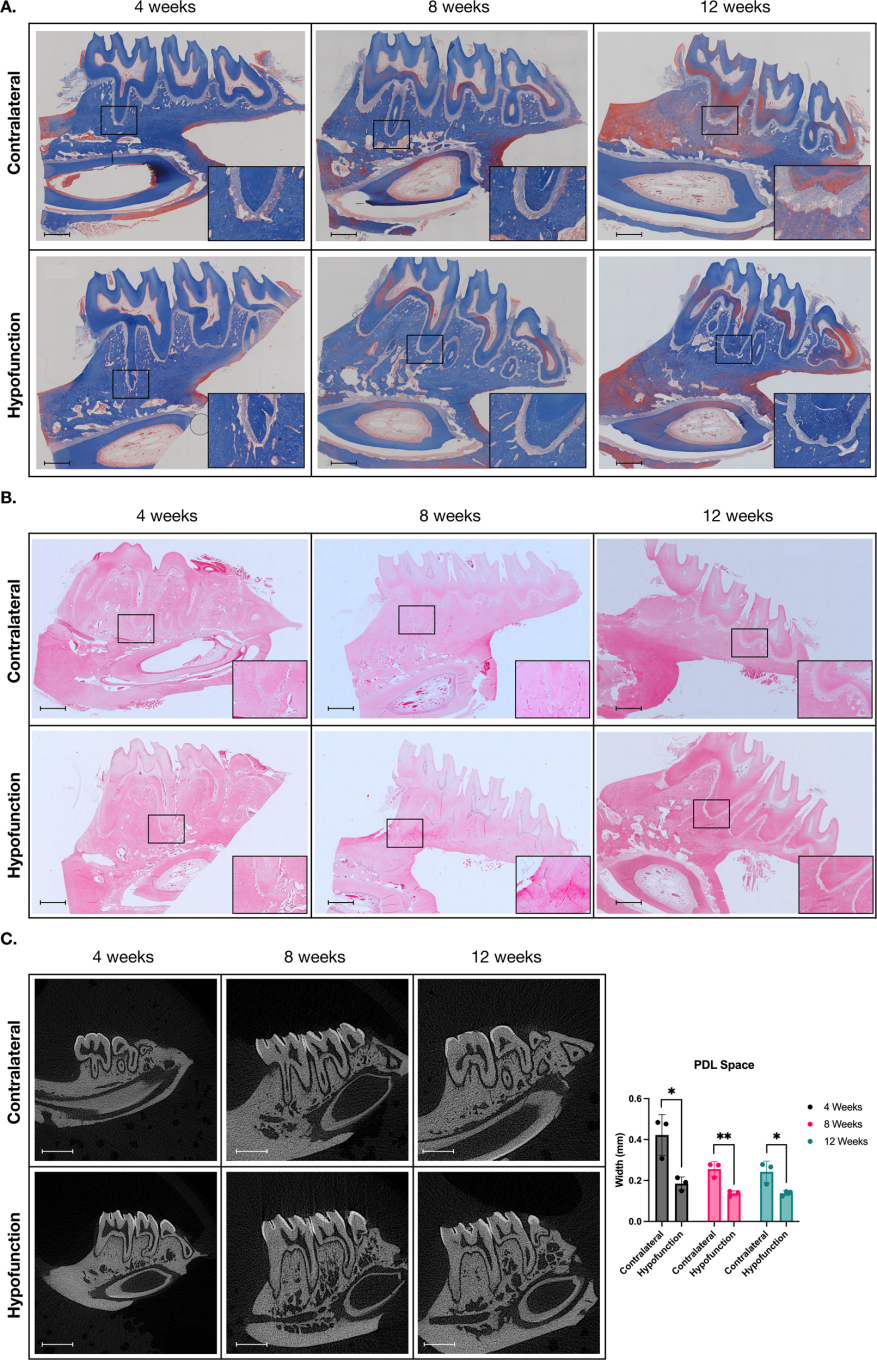
The Mechanical forces are essential for maintaining PDL integrity. The paraffin sections of the mandibular right molars (hypofunction tooth) and mandibular left molars (contralateral tooth) were stained for Masson’s trichrome (**A**) and H&E (**B**). **C** The micro-CT imaging showed the whole structure of the jawbone and teeth based on hydroxyapatite (HA) at 1200 mg HA/cc. The distance between cementum and alveolar bone (PDL space) is also shown in **C**. Data are presented from three biological replicates using samples obtained from three different rats. *p < 0.05 and **p < 0.01 compared to the control (cotralateral). The scale bar represents 1000 μm.

Figure 2A, B shows the entire structure of the rat mandible, with the three molars stained with Masson’s trichrome and H&E. A narrowing of the PDL space with an irregular and loose arrangement of collagen fibers was observed in the PDL of the hypofunctional tooth. This phenomenon was seen at all time points following extraction (Fig. 2A, B). This was confirmed with micro-CT scanning analysis (Fig. 2C). These findings suggest that occlusal force from the opposing tooth is required to maintain the width of the PDL.

### Changes of extracellular matrix protein expression in PDL of hypofunctional teeth

**Fig. 3:**
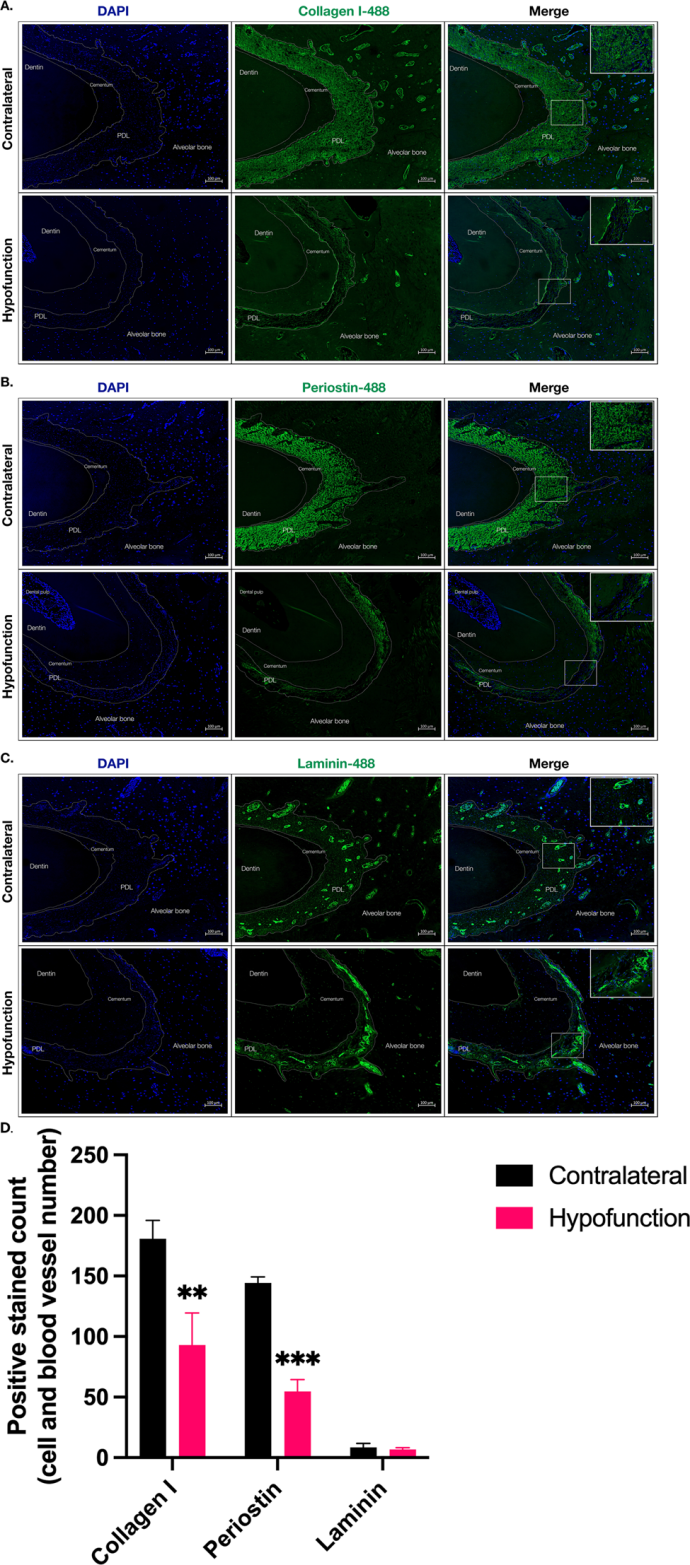
The effect of mechanical forces on ECM components of PDL at the apical root. The paraffin sections of the mandibular right molars (hypofunction tooth) and mandibular left molars (contralateral tooth) were stained with the antibody specific to collagen I, POSTN, and laminin. The photomicrographs were taken at the region of the apical root. (**A**) collagen I (**B**) POSTN, and (**C**) laminin. All targets were labeled with green fluorescent dye; AlexaFluor-488. DAPI was used for nuclei staining. Scale bar represents 100 μm. **D** The number of collagen I, POSTN positive ECM, and laminin-positive blood vessels. **p < 0.01 and ***p < 0.001 compared to the control (contralateral).

**Fig. 4:**
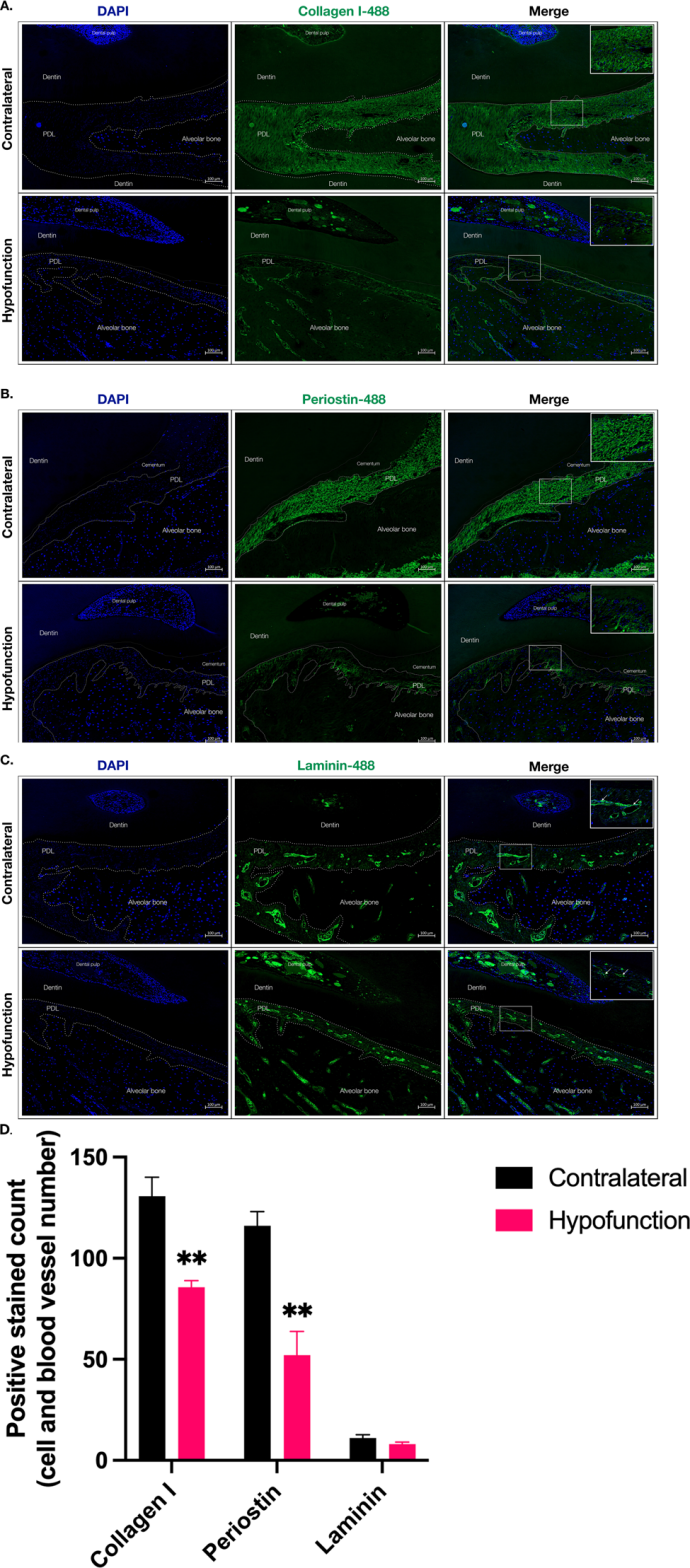
The effect of mechanical forces on ECM components of PDL at the middle root. The paraffin sections of the mandibular right molars (hypofunction tooth) and mandibular left molars (contralateral tooth) were stained with the antibody specific to collagen I, POSTN, and laminin. The micrographs were taken from the middle root. **A** collagen I (**B**) POSTN, and (**C**) laminin. All targets were labeled with green fluorescent dye; AlexaFluor-488. DAPI was used for nuclear staining. Scale bar represents 100 μm. **D** The number of collagen I, POSTN positive ECM, and laminin-positive blood vessels. **p < 0.01 compared to the control (contralateral).

Immunolocalization of various ECM proteins revealed that collagen I, POSTN, and laminin. Both collagen I and POSTN were uniformly expressed in the PDL of the contralateral side, while the expression was strongly decreased in the hypofunctional PDL at the apical (Fig. 3A, B) as well as middle root of mandibular molars (Fig. 4A, B). The quantification of positive cell and blood vessel count is presented in Figs. 3D and 4D. In the PDL of contralateral teeth, laminin was uniformly expressed in the laminae basalis of perivascular structures. There was no difference in laminin expression between the hypofunctional and contralateral side of both apical and middle roots of molars (Figs. 3C and 4C). Yet, the thickness of laminin-positive laminae basales decreased at the middle root of the hypofunctional molars (Fig. 4C), but such a decrease was not found in the PDL of the apical root (Fig. 3C). However, the number of laminin-positive blood vessels within the PDL space was not different (Figs. 3D and 4D). Our findings suggested that deficiency of mechanical force could reduce the expression of collagen I and POSTN with less laminin-positive perivascular structures in the PDL at the middle root of hypofunctional molars.

## Discussion

The present study was conducted to investigate how biomechanical and occlusal forces can modulate expression and structural integrity of certain ECM proteins in PDL. In the in vivo model, the reduction of biomechanical force had a negative impact on the PDL as per the narrower PDL space with reduced collagen I and POSTN expression. This was supported by the observation that POSTN expression was increased in vitro PDLSC culture in response to mechanical compressive and shear forces.

ECM is an extracellular complex structure of the tissue that primarily receives and transmits mechanical force stimuli. Matrix components usually bind to cell surface receptors, which modulate specific cellular activities through cell-ECM interaction [28]. The most abundant component of PDL-ECM is collagen type I, as seen by the significant accumulation of collagen I in PDL space (Fig. 3A). Disorientation of collagen fibers, in combination with sparse and disorganized features, was observed in hypofunctional teeth.

Collagen fibers serve as the main structural ECM component that perceives mechanical stresses and provides mechanical stability, elasticity, and structural strength [29, 30]. The densely arranged collagenous tissue such as tendon or PDL responds to the mechanical force in dualistic mechanisms in tissue remodeling; by destroying an existing tissue, or by forming a new tissue [10]. It has been shown that proper mechanical stimulation increases collagen formation, while insufficient stimulation led to collagen degradation at both molecular and physical levels [31]. In humans, mechanical stress on the patella tendon and quadriceps muscle increased collagen synthesis. Moreover, there was a 2- to 3-fold increase in collagen production within 24 h after exercise and is maintained for up to 70–80 h [32]. Alike those findings, we found an increase in the expression of collagen I protein from the force-induced PDLSCs and a dense arrangement of PDL tissue of the normal occlusal tooth of the in vivo model. On the other hand, static mechanical loading on 3-dimensional collagen-based dermal fibroblasts resulted in 2.5 to 13-fold increased expression of MMP-2, a type IV collagenase and gelatinase A, responsible for breakdown of denatured collagen [12]. In our study, the presence of collagen I in the periodontal space appeared markedly reduced in hypofunctional teeth. These findings suggest that the proper balance of biomechanical forces influences collagen structure.

POSTN is a matricellular protein that is known to be highly expressed in the PDL matrix [16]. This matrix protein was proposed to regulate tooth and bone remodeling, especially under mechanical stress due to its expression on sites that are generally exposed to mechanical loading by mastication or physical exercise [33, 34]. According to our in vitro findings, mechanical stimuli promoted production of POSTN in PDLSCs, indicating the relevance of POSTN in PDL responses to mechanical forces. In connective tissue, POSTN binds to type I collagen fibrils and control the process of collagen fibrillogenesis in mice [13]. These mice had decreased collagen crosslinking and collagen fibril diameter when POSTN was absent, indicating the relationship between POSTN and the collagen fibril turnover process. Our study revealed that collagen I expression was decreased in PDLSCs under both compression and shear forces. This expression pattern contrasts with the one of POSTN under the same conditions. In applying stress to a single cell layer without support from the ECM, direct mechanical force stimuli probably change collagen biosynthesis, which is reflected in a decrease of collagen I expression. The use of additional supplements, such as ascorbic acid, to enhance the thickness of PDLSCs-ECM layer may improve the in vitro study and make it more relevant to the in vivo investigation [35]. However, these findings may also suggest that POSTN may maintain PDL homeostasis by modulating collagen turnover during mechanical force stimulation.

Furthermore, laminin is another important component of the ECM, generally found in all laminae basalis [19, 36]. In this study, laminin was highly expressed by laminae basalis of blood vessels in the PDL. This expression was slightly decreased at the middle root of hypofunctional teeth. This suggests a negative impact of low-intensity occlusal forces on the vascular structure of the PDL. Apoptosis of vascular endothelial cells with subsequent atrophic change of vascular structure has been reported in PDL of hypofunctional tooth [37]. Laminin is an ECM protein that due to its localization adjacent to the vascular endothelium is often exposed to fluid shear force and acts as a ligand for mechanical force-sensitive receptors such as integrins [19, 20]. Moreover, laminin has been shown to facilitate SF regulation of β-catenin signaling in colon cancer cells [21]. Thus, this protein is potentially associated in mechanical force responses, particularly under SF stimulation. A previous study had demonstrated that laminin can facilitate the alignment of endothelial cells in the direction of flow, thereby improving their ability to withstand and respond to shear force [38]. In our investigation, no significant changes in the laminin expression pattern were found. It has been noted, however, that once the occlusal force is applied to a tooth, a range of forces and magnitudes are created at various root locations. Under all loading situations, the largest root biomechanical stress occurred in the cervical third of the root surface and not in the apical one [39]. It is possible that variations in the laminin expression pattern are associated with the shear force-dominant location at the level of the root surface. Though unveiling this association will require further investigations.

In summary, it is possible that the interaction between POSTN and collagen I promoted PDL-ECM homeostasis during biomechanical force stimulation. The reduction of POSTN and collagen I subsequently interfered with ECM structural integrity, reflected as narrowing PDL space under low-intensity of occlusal forces. Yet, it is interesting that no visible structural change occurred in the periodontium adjacent to the contralateral molars, the teeth that were presumably hyperfunctional. As opposed to hypofunctional teeth, hyperfunction or parafunctional periodontium typically manifest as larger ligament space but decreased alveolar bone mass compared to normal function teeth [40]. In hyperfunctional habits, repeated or excessive occlusal force is applied on tooth and supporting which exceed the physiologic limits of the tissue tolerance and subsequently leads to occlusal trauma and destruction of periodontal tissue [41]. The occlusal trauma can be generated by an excessive occlusal force which is classified as either primary or secondary depending on the condition of the connective tissue attachment and bone levels. The primary stage includes normal connective tissue attachment and normal bone levels, which were demolished in secondary stage [42]. In our study, there were no signs of alveolar bone and loss of PDL structure in the contralateral mandible during 12 weeks of the study which may imply as the primary stage of occlusal trauma with no loss of connective tissue occurs.

Our findings reveal that both the type and magnitude of a biomechanical/occlusal force are essential to preserve ECM stability and strength in the PDL via the modulation of two essential ECM proteins, collagen I and POSTN. The healthy PDL-ECM may provide an immobilized niche environment for PDLSCs to maintain their stemness and provide PDL tissue integrity. Studies have indicated that occlusal hypofunction is one of the major factors leading to dental root and bone resorption [3, 4]. It is possible that strengthening the PDL-ECM by targeting matrix proteins might be an alternative approach for maintaining ECM homeostasis and preventing the destruction of periodontal tissue caused by occlusal hypofunction, especially in cases of unknown etiology. Future studies are necessary to further investigate the mechanisms of ECM proteins that regulate the PDL structural integrity.

## Conclusion

Our findings revealed that a deficiency in occlusal force interferes with the deposition of collagen I, POSTN, and laminin and highlight the importance of mechanical forces in modulating ECM composition to preserve periodontal tissue homeostasis.

## Supplementary information

Supplymentary materials

## Data Availability

The data supporting the findings of this study are available in both the article and its supplementary materials. Additional datasets are available from the corresponding author upon reasonable request.
