# Supplementary material for: Alteration of extracellular matrix proteins in atrophic periodontal ligament of hypofunctional rat molars
Source: BDJ Open. 2023 Jul 18;9:31. doi: 10.1038/s41405-023-00155-7 (PMC10353993; doi:10.1038/s41405-023-00155-7)
Supplement: Supplementary file 1 — Supplymentary materials [file 41405_2023_155_MOESM1_ESM.docx]

**Supplementary material**


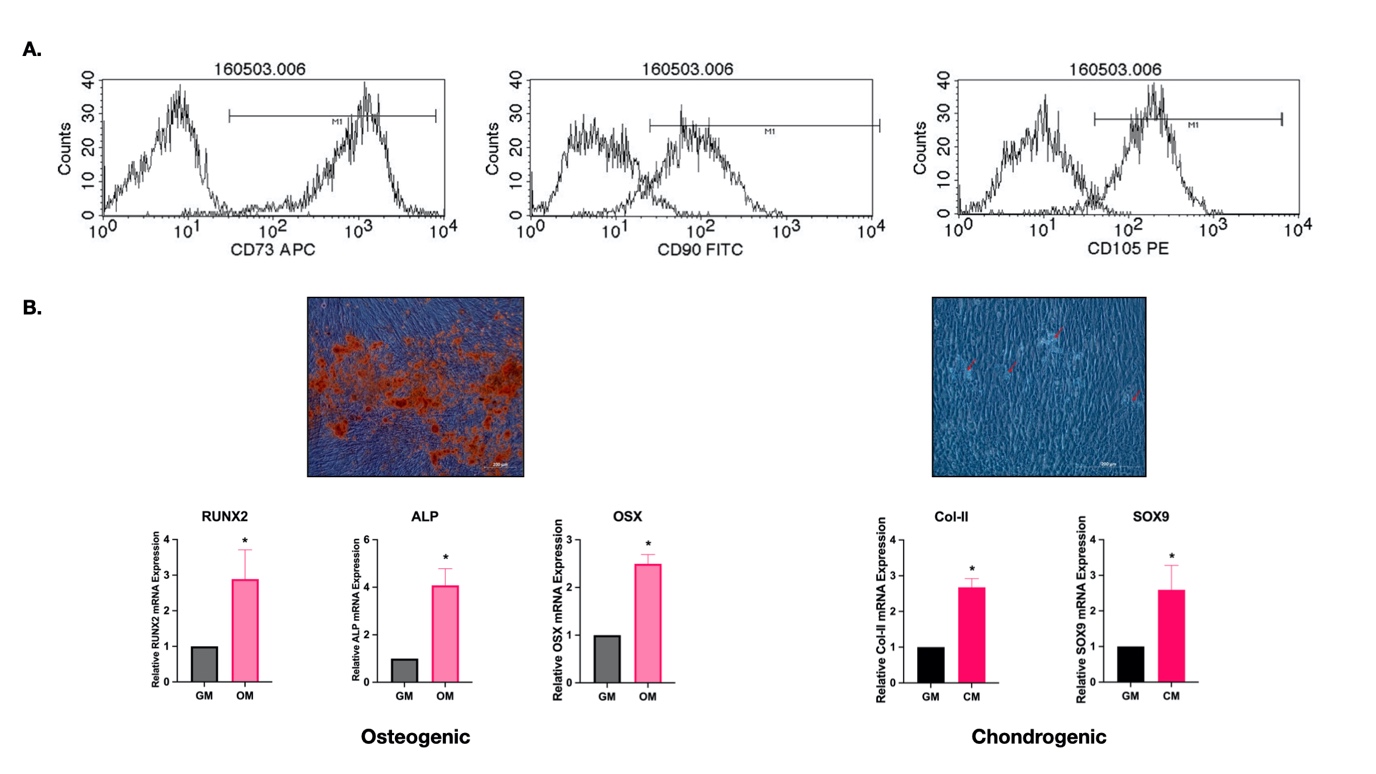


**Supplementary Figure 1. Human PDLSCs characterization.**

PDLSCs were stained with surface markers CD73, CD90, and CD105 antibodies. **A.** Showing the overlayed histogram of each marker compared to isotype control. **B.** The osteogenic and chondrogenic differentiation: PDLSCs osteogenic capacity was demonstrated by Alizarin red staining after 14 days induction in the osteogenic induction medium (OM) and osteogenic marker mRNA expression (RUNX2, ALP and OSX) after 5 days induction in OM compared to general medium (GM). The PDLSC's chondrogenic capacity was demonstrated by Alcian blue staining after 14 days of induction in the chondrogenic induction medium (CM) and osteogenic marker mRNA expression (Col-II and SOX9) after 5 days of induction in OM compared to GM. * P<0.05 compared to GM.

**A.**

**
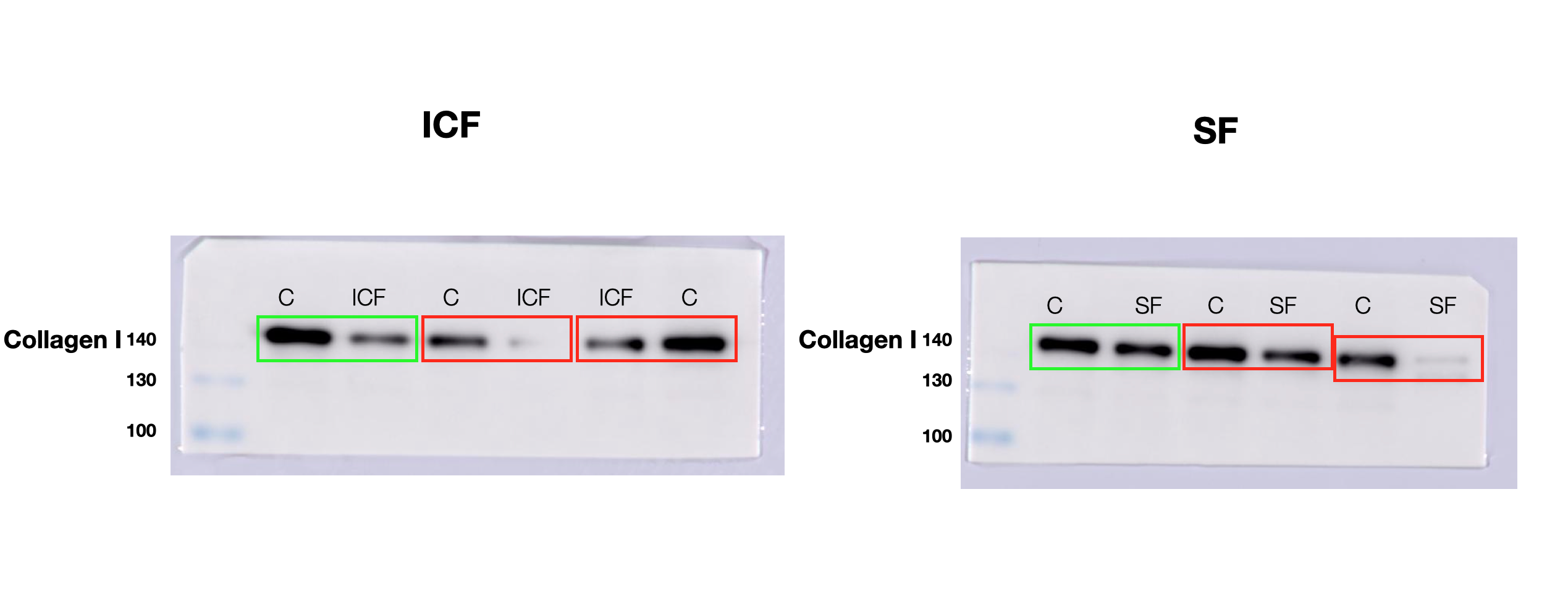
**

**B.**

**
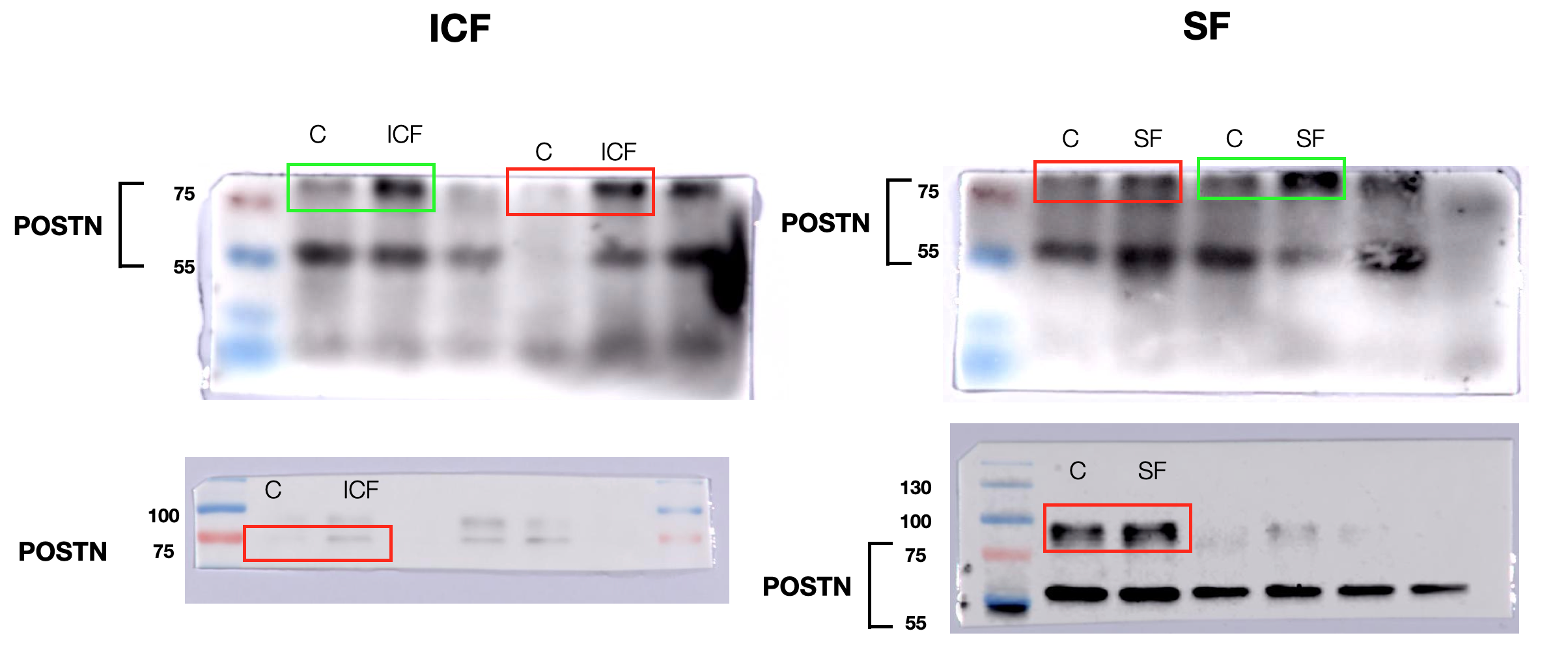
**

**Supplementary Figure 2. Uncropped WB data.**

(A) Uncropped Collagen I WB analysis image, protein size is 140 kDa. (B) Uncropped POSTN WB analysis image, protein size is 75 kDa. The Green square was used as the representative band. The other two replicates were shown in the red square, all three replicates have been used for the quantification. Non-labeled lanes were the cell’s condition, which was not part of this experiment.
